# Melatonin in Traumatic Brain Injury and Cognition

**DOI:** 10.7759/cureus.17776

**Published:** 2021-09-06

**Authors:** Brian Blum, Shivani Kaushal, Sara Khan, Jae H Kim, Clara L Alvarez Villalba

**Affiliations:** 1 Psychiatry, Aventura Hospital and Medical Center, Aventura, USA; 2 Medicine, Nova Southeastern University Dr. Kiran C. Patel College of Allopathic Medicine, Davie, USA; 3 Psychiatry, Nova Southeastern University Dr. Kiran C. Patel College of Allopathic Medicine, Davie, USA

**Keywords:** traumatic brain injury, melatonin, inflammation, alzheimer's disease, cl psychiatry, psychiatry, neurology, dementia, geriatric psychiatry, brain awareness

## Abstract

Traumatic brain injury (TBI) is a leading cause of long-term disability and mortality in young adults. The devastating effects of TBI on emotion regulation, executive functioning, and cognition have been well-established, and recent research links TBI as a risk factor for neurodegenerative diseases such as Alzheimer’s disease. Despite an increased focus on the long-term cognitive dysfunction associated with TBI, research into potential treatments has not yet generated consistent successful results in human subjects. Many foundational studies have analyzed the cellular and molecular events involved in the inflammatory and healing processes following TBI, enhancing our understanding of the mechanisms that may contribute to the progression of dementia and cognitive decline in these patients. In this review, we will discuss the emergent research on melatonin within the framework of neuroinflammation and oxidative stress resulting from TBI and possibly preventing further sequelae such as Alzheimer’s disease.

A literature review was conducted using standard search strategies to query the PubMed database. The following search terms were used with qualifiers of various combinations: TBI, traumatic brain injury, melatonin, treatment, dementia, Alzheimer’s, cognition, and neurodegeneration. Selected studies included meta-analyses, literature reviews, and randomized controlled trials (RCT) that evaluated melatonin’s role as a potential therapy to prevent post-TBI neurodegeneration, specifically the development of dementia and deficits in memory and cognition. Three independent reviewers assessed all articles for eligibility. After assessment for eligibility, 11 total studies were included.

Much of the available data on melatonin in TBI has highlighted its significant neuroprotective and antiinflammatory effects, which can be significant in fighting against the neuroinflammatory processes indicated in neurodegeneration. In animal models, immunohistochemistry and histopathology have allowed researchers to study measures of cell injury such as inflammatory cytokines, edema, and markers of oxidative stress. Though the effects of melatonin in TBI appear to be mediated through mostly indirect mechanisms on inflammatory processes, some research has explored potential mechanisms that could be specific to melatonin.

Animal model studies support that melatonin treatment after TBI significantly improves cognition and behavioral outcomes. However, clinical studies with human subjects are scarce. Beyond the apparent general antiinflammatory and antioxidant actions of melatonin, a review of the evidence identified some preliminary research that has suggested the significance of melatonin receptors specifically in TBI. While there is some evidence to suggest that melatonin is able to reduce post-TBI cognitive decline as measured by subject performance on memory tasks, there is a lack of longitudinal data on whether melatonin decreases the risk of developing dementia after TBI. Considering melatonin therapy’s promising preclinical data, favorable safety profile, and accessibility, further studies are warranted to assess the effects of melatonin as a post-TBI therapy on human subjects.

## Introduction and background

Traumatic brain injury (TBI) is a leading cause of long-term disability and mortality in young adults. The devastating effects of TBI on emotion regulation, executive functioning, and cognition have been well-established [1,2]. Recent literature similarly suggests that TBI is a risk factor for neurodegenerative diseases such as Alzheimer’s disease [3,4]. Despite an increased focus on the long-term cognitive dysfunction associated with TBI, research into potential treatments has not yet generated consistent successful results in human subjects. However, a plethora of studies has analyzed the cellular and molecular events involved in the inflammatory and healing processes following TBI, enhancing our understanding of the mechanisms that may contribute to the progression of dementia and cognitive decline in these patients. Studies have shown that persistent neuroinflammation with chronic microglial activation may contribute to the future development of neurodegenerative disease following TBI. Moreover, axons are particularly vulnerable to injury due to TBI-induced white matter degeneration, and the damaged axons may serve as a reservoir for amyloid precursor protein (APP) and beta-amyloid (Aβ) [3,4]. In addition, chronic post-TBI neuroinflammation may result in impairment of the ubiquitin-proteasome pathway for protein degradation, resulting in increased Aβ and p-tau (phosphorylated tau) deposition [3]. It has also been found that TBI depletes intracellular energy stores, causing failure of ATP-dependent ion pumps, which results in membrane depolarization and subsequent glutamate release. Glutamate incites neuronal cell death via excitotoxicity, which involves the release of reactive oxygen species (ROS) [5,6]. Therefore, it is thought that therapies promoting a decrease in neuroinflammation and oxidative stress may be able to prevent the development of neurodegenerative diseases such as Alzheimer’s disease after TBI. 

There are currently no Food and Drug Administration (FDA)-approved therapies for treating TBI. Due to the risk of post-TBI neurodegenerative disease, it is critical to investigate potential therapies that may prevent adverse outcomes in these patients. Melatonin, a naturally occurring hormone that crosses the blood-brain barrier and binds receptors in the central nervous system has been found to have neuroprotective effects that may reduce symptom burden after TBI [7]. Various studies have shown that melatonin exerts its neuroprotective action through its antiinflammatory and antioxidant capabilities, which may contribute to reduced risk of post-TBI secondary injury and subsequent functional deficits. Because such neuroprotection has been theorized to reduce the cognitive deficits apparent after TBI, recent research has investigated the effect of melatonin supplementation in post-TBI cognitive processes. In an attempt to more clearly characterize this supplement’s specific role in cognition, we have examined the most recent literature on this nascent and developing topic. In this review, we will summarize the role of melatonin in preventing post-TBI neurodegeneration, particularly focusing on melatonin’s potential to reduce the risk of cognitive impairment after TBI. 

Melatonin, or 5-methoxy-N-acetyltryptamine, is most colloquially known as a sleep aid due to its availability as an over-the-counter supplement for sleep. Synthesized at night and secreted by the pineal gland, melatonin configures the circadian rhythm and has many pleiotropic effects, including antiinflammatory, antioxidant, and cell cycle-modulating properties [8]. The nycthemeral rhythm of endogenous melatonin secretion is based on the sun. Light is relayed from photosensitive ganglion cells in the retina via the retinohypothalamic tract as an inhibitory signal to the hypothalamus, suppressing the suprachiasmatic nuclei (SCN) and melatonin production during the day. Thus, melatonin is known as “the darkness hormone” and mediates entrainment to our external environmental cues. When administered at night, artificial light of sufficient intensity and duration suppresses melatonin production and disrupts melatonin rhythm [9].

The pathway continues as the SCN synapses with preganglionic cell bodies of the superior cervical ganglia (SCG) of the sympathetic chain in the upper part of the cervical spinal cord. The SCG therein send projections to the pineal gland and release nocturnal norepinephrine, the major regulatory neurotransmitter of the pineal gland. Melatonin production in the pineal gland is dependent on circulatory tryptophan, where it is taken up and transformed to serotonin. The binding of norepinephrine to adrenergic β1 receptors activates pineal adenylate cyclase, increasing cyclic AMP (cAMP) and de novo synthesis of serotonin-N-acetyl transferase (NAT), the rate-limiting enzyme for melatonin synthesis. Drugs that increase synaptic catecholamine availability, such as MAO inhibitors or tricyclic antidepressants, reinforce melatonin secretion. Conversely, drugs that decrease synaptic catecholamine availability, clonidine, and a-methyl-para-tyrosine, and β1-adrenergic receptor blockers suppress nightly melatonin secretion [10].

There is no pineal storage of melatonin. As it is released in the circulation, it gains access to various fluids, tissues, and cellular compartments by means of its high lipid and water solubility (octanol/water coefficient of partition≈13). The liver primarily metabolizes melatonin, where it is first hydroxylated, then excreted in urine as sulfate and glucuronide conjugates. The maximum plasma level is around 03:00-04:00 a.m. for most chronotypes, with undetectable or low diurnal levels, reflecting the greatest nycthemeral amplitude change observed for a hormone [10].

Melatonin provides the body with night information and is considered to be the organizer of circadian rhythm, facilitating core temperature and cortisol cycles. Two high-affinity G protein-coupled membrane receptors, MT1 and MT2, have been presently identified in humans throughout the body, including the brain, retina, cardiovascular system, and skin. Melatonin facilitates sleep predisposition through vasodilation via MT2 receptor [11]. Physiological doses of melatonin inhibit the in vitro ACTH-stimulated cortisol production [12]. It also theorized that melatonin is a stabilizing factor within other physiological functions, including immune, antioxidant defenses, hemostasis, and glucose regulation. Beyond its membrane receptor activation, the small lipophilic melatonin can pass through biological membranes, functioning as a ligand for the orphan nuclear hormone receptor superfamily RZR/RO and influencing its pro-apoptotic effect on cancer cells [13]. As an antioxidant, melatonin is more potent than vitamin E and directly scavenges radicals. It further elevates its antioxidant role by raising levels of several antioxidative enzymes, including superoxide dismutase, glutathione peroxidase, and glutathione reductase, and inhibiting pro-oxidative enzyme nitric oxide synthase [14]. Melatonin scavenging of oxidative radicals in lymphoid cells is thought to have an immunomodulatory impact on inflammation and other inflammatory disease processes, such as traumatic brain injury and its sequelae.

## Review

Methods

A literature review was conducted using standard search strategies to query the PubMed database. The following search terms were used with qualifiers of various combinations: TBI, traumatic brain injury, melatonin, treatment, dementia, Alzheimer’s, cognition, and neurodegeneration. Selected studies included meta-analyses, literature reviews, and randomized controlled trials (RCT) that evaluated melatonin’s role as a potential therapy to prevent post-TBI neurodegeneration, specifically the development of dementia and deficits in memory and cognition. Three independent reviewers assessed all articles for eligibility. After assessment for eligibility, 11 total studies were included. 

Results and discussion

The present study on the role of melatonin for dementia in TBI is sparse. A PubMed database keyword search for studies containing all three keywords of “TBI,” “melatonin,” and “dementia” returned zero results, while a search with the keywords “TBI,” “melatonin,” and “Alzheimer’s” returned just one study. However, broader searches including keywords such as “cognition” and “neurodegeneration” yielded more results that suggested the presence of some foundational data about melatonin’s potential roles in cognitive processes post-TBI. The studies are presented in Tables 1, 2.

**Table 1:** Previous reviews on melatonin and TBI TBI: traumatic brain injury; NFkB: nuclear factor kappa light chain enhancer of activated B cells; AP1: activator protein 1; CNS: central nervous system; ROS: reactive oxygen species; SCI: Science Citation Index; NOS: nitric oxide synthases; CSF: cerebrospinal fluid

Citation	Title	Study design and participants (n) if applicable	Key findings
Barlow et al., 2019 [15]	Melatonin as a Treatment after Traumatic Brain Injury: A Systematic Review and Meta-Analysis of the Pre-Clinical and Clinical Literature	Meta-analysis and systematic review 17 studies (15 pre-clinical, 2 clinical) met inclusion criteria	Preclinical studies (15): Melatonin treatment → favorable effect on overall neurobehavioral outcome (standardized mean difference [SMD] = 1.51), neurological status (SMD = 1.35), and motor outcomes (SMD = 1.93) Overall, melatonin improved performance on a memory-based cognitive task by a standardized mean difference of 1.16 Melatonin decreased contusion size by SMD = 2.22 Clinical studies (2): N = 7 adult males; double-blind randomized crossover trial: No effect of melatonin treatment on sleep or neuropsych parameters compared with active comparison to amitriptyline N = 12 children with post-concussion syndrome & post-traumatic headaches following TBI; uncontrolled, open-label retrospective cohort: 75% had >50% improvement in # of headaches
Osier et al., 2018 [7]	Melatonin as a Therapy for Traumatic Brain Injury: A Review of Published Evidence	Literature review on melatonin as a treatment for TBI 22 articles after applying exclusion criteria	Melatonin (MEL) therapy provides neuroprotective benefits after TBI through multiple mechanisms, including the potentiation of antioxidants, inhibition of late-phase activation of NFkB, decreased AP-1 levels, and reduced apoptosis, resulting in subsequent attenuation of functional deficits MEL significantly reduces contusion volume with major effects during the night, which may be due to reduction of early free radical formation The dose-response of MEL appears to follow a bell-shaped curve with a short therapeutic window where the middle dose confers the most benefit Most studies in this review support the conclusion that the use of MEL as a method for treatment of TBI, if used under appropriate conditions at an appropriate dosage, can be beneficial The majority of studies utilized adult male rats as subjects and examined acute, rather than chronic, outcomes after TBI
Esposito and Cuzzocrea, 2010 [16]	Antiinflammatory Activity of Melatonin in Central Nervous System	Literature review on the effect of melatonin in traumatic CNS injury and neurological diseases including dementia	Glucocorticoids provide beneficial effects after acute CNS injury in humans and experimental animals. Important data show that glucocorticoids may have a disease-modifying effect in addition to anti-inflammatory actions Glucocorticoids are among a variety of endogenous compounds that have been suggested to influence melatonin production Among animal models of Alzheimer’s disease, melatonin has been found to reduce ROS, block apoptosis, have antiinflammatory effect, improve spatial memory, and protects wortmannin-induced tau hyperphosphorylation
Naseem and Parvez, 2014 [17]	Role of Melatonin in Traumatic Brain Injury and Spinal Cord Injury	Literature review on the protective role of melatonin in central nervous system injuries including TBI and spinal cord injury (SCI)	The neuroprotective functions of melatonin in TBI and SCI include its ability to reduce brain edema, decrease late-phase activation of NFkB, decrease AP-1 to basal levels, regulate inducible NOS, and increase the activity of superoxide dismutase and glutathione peroxidase, which protect against oxidative stress Both endogenous and exogenous melatonin have been found to provide neuroprotection. The level of endogenous melatonin in the CSF increases after TBI. There is limited data available on the adverse effects of melatonin, but recent studies indicate that melatonin may generate intracellular ROS via a reduction of intracellular glutathione activity in U937 cells

**Table 2:** Review of studies on melatonin in cognition after TBI rmTBI: repeat mild traumatic brain injury; Nrf2: nuclear factor erythroid 2-related factor 2; CREB: cAMP-response element-binding protein; AMP: adenosine monophosphate; CA1: carbonic anhydrase 1; BACE-1: beta-site amyloid precursor protein cleaving enzyme 1; APP: amyloid precursor protein; NIR iLED: near-infrared intranasal light-emitting diode; PTSD: post-traumatic stress disorder; MDA: malondialdehyde; SOD: superoxide dismutases; ROS: reactive oxygen species; RCT: randomized controlled trial; GSH/GSSG: reduced glutathione/oxidized glutathione ratio; PCR: polymerase chain reaction; IHC: immunohistochemistry; TNF-AIP3: tumor necrosis factor-α-induced-protein 3; CCI: controlled cortical impact; BBB: blood-brain barrier

Citation	Title	Study design and participants (n) if applicable	Evidence of improved cognition or reduced inflammation with melatonin post-TBI	Key findings
Rehman et al., 2019 [18]	Neurological Enhancement Effects of Melatonin against Brain Injury-Induced Oxidative Stress, Neuroinflammation, and Neurodegeneration via AMPK/CREB Signaling	RCT using male C57BL/6N mice divided into 4 groups with n = 15. The 4 groups included a saline-treated control group (control), repetitive mild traumatic brain injury (rmTBI) group, repetitive mild traumatic brain injury plus daily intraperitoneal (i.p) injection of 20 mg/kg melatonin (rmTBI plus Mel), and a sham-treated group (Mel) that received daily i.p injection of 20 mg/kg melatonin but was without TBI	Yes (cognition and inflammation)	Melatonin resulted in higher levels of the antioxidant Nrf2 in the brains of the rmTBI plus Mel-treated mice compared to the rmTBI mice not treated with melatonin, suggesting melatonin exerted antioxidant activity. Expression levels of cyclooxygenase-2 and inducible nitric oxide synthase were significantly inhibited with melatonin therapy in the rmTBI plus Mel-treated group, suggesting a melatonin-induced decrease in neuroinflammation. The rmTBI plus Mel-treated mice had increased expression of the pro-survival CREB proteins in the cortex and hippocampal CA1 region compared to the rmTBI mice. Melatonin reduced apoptotic cell death and lesion volume Melatonin significantly reduced the expression levels of the Alzheimer’s disease markers BACE-1, APP, and Aβ in the rmTBI plus Mel-treated mice. Melatonin was found to rescue memory dysfunction. In the Y-maze test, the rmTBI plus Mel mice displayed a higher percentage of spontaneous alternation behavior, suggesting improved cognitive performance compared to the rmTBI mice
Naeser et al., 2016 [19]	Transcranial, Red/Near-Infrared Light-Emitting Diode Therapy to Improve Cognition in Chronic Traumatic Brain Injury	Trial of LED therapy post-TBI with pre-and post-testing with measures of executive function, memory, etc. Review of literature on photobiomodulation (PBM) for TBI patients	Yes (cognition)	PBM targets injured brain cells and may improve the function of brain networks that regulate attention, executive function, memory, emotions, and behavior. Ongoing studies: NIR iLED hypothesized to deliver photons to the hippocampus; red 633 nm iLED believed to increase melatonin (hypothesize that some NIR photons that are delivered into the nose can reach medial temporal lobe structures, including the hippocampus and the adjacent perirhinal, entorhinal, and parahippocampal areas). Preliminary results: significant improvements in executive function, verbal memory, attention, verbal fluency; sustained at 12 wks post-treatment; sleep efficiency and average total sleep time increased. Conclusion from review: significant improvements in measures of executive function, verbal learning, and memory; also fewer reported PTSD symptoms
Ge et al., 2020 [20]	Effect of melatonin on regeneration of cortical neurons in rats with traumatic brain injury	Sprague-Dawley rats (n=36) were randomly divided into sham, TBI+vehicle, and TBI+melatonin groups. Cerebral blood flow and cognitive function observed via laser Doppler flowmetry and by Morris water maze testing, respectively Serum malondialdehyde (MDA) and superoxide dismutase (SOD) levels + IHC were used to observe neurons and apoptotic cells	Yes (cognition and inflammation)	Cognitive function of rats in the TBI+melatonin group was significantly higher than that in the TBI+vehicle group Cerebral blood flow in the TBI+melatonin group was higher, but not significantly, than that of the TBI+vehicle group at one, 12, 24, and 48 h post-injury MDA and SOD levels higher in TBI+melatonin group when compared to those in TBI+vehicle group but similar when compared to those in TBI+sham group. Number of apoptotic cells lower in the TBI+melatonin group than in the TBI+vehicle group
Ozdemir et al., 2005 [21]	Effect of Melatonin on Brain Oxidative Damage Induced by Traumatic Brain Injury in Immature Rats	RCT using rats divided into three groups: vehicle-treated TBI group (TBI, n=7), melatonin-treated TBI group (TBI+Mel, n=7), and control group (n=7) receiving sham surgery	Yes (inflammation)	ROS induce lipid peroxidation and play a key role in mediating TBI because the brain is particularly vulnerable to oxidative damage due to its high rate of O_2_ consumption, high production of free radicals, and high levels of transition metals. Melatonin, a free radical scavenger, administered as a single dose of 5 mg/kg prevented TBT-induced increase in thiobarbituric acid reactive substances (TBARS) levels in both non-traumatized and traumatized brain hemispheres
Rui et al., 2021 [22]	Deletion of ferritin H in neurons counteracts the protective effect of melatonin against traumatic brain injury-induced ferroptosis	RCT of ferritin H knockout mice and WT mice were randomly divided into groups (each group n=6-8) for various combinations of melatonin/sham tx, timeline for TBI, etc.). Ferroptosis degree and outcomes measured by Western blot analysis, PCR, GSH/GSSG assay, iron assay kit, IHC, Prussian blue staining, etc.	Yes (inflammation)	Evidence supports TBI-induced ferroptosis may explain the pathophysiological process of TBI. Neuron-specific ferritin H may play a role in melatonin’s neuroprotection and this study showed that neuron-specific ferritin H knockout mice were more susceptible to ferroptosis after TBI and neuroprotection by melatonin was largely abolished in the knockout mice compared to control
Bao et al., 2019 [23]	Silencing of A20 Aggravates Neuronal Death and Inflammation After Traumatic Brain Injury: A Potential Trigger of Necroptosis	RCT of rats into three groups: control (n=5), sham (n=5), and CCI/TBI group (n=30). Assessment of hippocampus and cortical tissue using western blot, rt-PCR, and immunoblotting	Yes (inflammation)	A20, or TNF-AIP3, regulates necroptosis and inflammation after TBI in rat model of TBI and lack of A20 led to aggressive necroptosis and attenuated anti-necroptotic effects of necrostatin-1 and melatonin. Additionally, melatonin alleviated necroptosis. Another study found: once-daily injection of P7C3-A20 for 30d improved BBB tight junction protein expression, ceased chronic axonal degeneration, and reduced brain infiltration of immunoglobulins in mice [24]
Osier et al., 2017 [25]	Brain injury results in lower levels of melatonin receptors' subtypes MT1 and MT2	RCT using 25 adult male Sprague Dawley rats, with 6 or 7 rats per group. Rats were randomly assigned to receive either TBI using controlled cortical impact or sham surgery, with humane euthanization occurring at either six or 24 hours after surgery for brain harvestation in order to detect melatonin receptor (MT1 and MT2) levels via western blotting	N/A	Both MT1 and MT2 levels were reduced in the frontal cortex at 24 hours and in the hippocampus at both six hours and 24 hours MT1 and MT2 levels decrease in a time point- and region-specific manner after TBI, which may alter the response to MEL therapy. Findings suggest the melatonergic system is implicated in TBI pathology and/or recovery

Much of the available data on melatonin in TBI has highlighted its significant neuroprotective and antiinflammatory effects, which are critical in fighting against the neuroinflammatory processes indicated in neurodegeneration and injury. As a physiological protective mechanism, the level of endogenous cerebrospinal fluid (CSF) melatonin increases in TBI patients to suppress the level of oxidants, and both endogenous and exogenous melatonin have been found to combat oxidative stress in the brain [17]. Immunohistochemistry and histopathology in animal models have allowed researchers to study measures of cell injury such as inflammatory cytokines, edema, and markers of oxidative stress. In one review of melatonin’s roles in central nervous system (CNS) injuries, melatonin was reported to reduce neuroinflammation and edema, decrease late-phase activation of nuclear factor-kappa light chain enhancer of activated B cells (NFkB), decrease activator protein 1 (AP-1) to the basal level, and increase the activity of superoxide dismutase and glutathione peroxidase, which protect cerebral tissue against oxidative stress [17]. Cerebrovascular injury may also be a major contributor to neuroinflammatory damage [5,6]. 

Persistent neuroinflammation may provide a link between a past TBI and the future development of neurodegenerative diseases [3,4]. This connection offers a strong basis for establishing the role of melatonin in cognitive decline after TBI due to its roles in inflammation and brain injury. As suggested by experiments investigating patterns of positron emission tomography (PET) ligand binding in TBI patients, increased microglial activity may persist long after TBI. Increased binding is correlated with more severe cognitive impairment, suggesting chronic inflammatory response, especially in subcortical regions [26]. In the only study specifically looking at melatonin’s role in neurodegeneration after TBI, researchers saw increased levels of ROS and malondialdehyde (a marker of lipid peroxidation) and reduced expression of the antioxidant protein nuclear factor erythroid 2-related factor 2 (Nrf2) in the brains of mice following repetitive mild TBI [18]. They also found treatment with melatonin reduced these effects, indicating the anti-inflammatory and antioxidant roles of melatonin. In the study, melatonin had further injury-reducing effects through decreased apoptotic cell death and lesion volume and a significant reduction in levels of expression of the Alzheimer’s disease marker proteins beta-site amyloid precursor protein cleaving enzyme 1 (BACE-1), APP, and Aβ proteins. Following melatonin treatment, these mice also had improved performance in the Y-maze and beam walking tests, which are clinical models for measuring cognition and motor function, respectively. Other performance tests of cognition used in animal studies of melatonin post-TBI included the Morris water maze and a battery of behavioral assessments including novel context mismatch and swim force tests. Measures of motor function and coordination include time on a wire grip, balance beam, and Rotarod apparatus [15]. Performance measures have all been shown to improve after melatonin administration post-TBI, suggesting a role of melatonin in preventing the neurodegenerative changes associated with TBI. 

Studies on adult mice have shown that melatonin administered at specific doses decreased lipid peroxidation levels and promoted antioxidant activity following TBI [7]. Melatonin appears to demonstrate similar neuroprotective effects in the pediatric population. In one study evaluating the effect of melatonin on oxidative damage induced by TBI in seven-day-old rat pups, results show that a single dose of melatonin at 5 mg/kg prevented an increase in levels of thiobarbituric acid reactive substances (TBARS), byproducts of lipid peroxidation [21]. A similar study by Ozdemir et al. in 2005 utilizing seven-day-old rats found that melatonin preserved hippocampal neurons following TBI and decreased deficits in spatial memory as identified by performance on a water maze task [7]. These results reinforce the idea that melatonin protects against secondary brain injury induced by ROS after TBI, which could attenuate the development of post-TBI cognitive decline in the future. 

Though the effects of melatonin in TBI appear to be mediated through mostly indirect mechanisms on inflammatory and oxidative processes, some research has explored potential mechanisms that could be specific to melatonin and its receptors. One study showed that melatonin receptors (MT1 and MT2) were less abundant in the frontal cortex and hippocampus of rats 24 hours following controlled impact TBI compared to after sham surgery [25]. The authors note that this finding, though it requires more elucidation, could potentially provide an explanation behind the wide range in the efficacy of melatonin therapy after TBI. 

Furthermore, in addition to generalized effects on inflammatory processes following TBI, melatonin appears to indirectly affect cognition through its other known physiological effects on sleep-wake cycles. For example, poor sleep patterns can disrupt normal processes of metabolite clearance that occur during sleep, leading to the accumulation of metabolites such as beta-amyloid and other neurotoxic waste products. Thus, some researchers have explored the effects of transcranial photobiomodulation (tPBM) for TBI patients as red (633 nm) intranasal light-emitting diode (iLED) is believed to increase melatonin [19]. NIR (near-infrared) photons are also used in tPBM, and photons delivered into the nose can reach medial temporal lobe structures, including the hippocampus and the adjacent perirhinal, entorhinal, and parahippocampal areas [19]. Preliminary results of tPBM in TBI patients have shown significant improvements in executive function, verbal memory, attention, verbal fluency, which were sustained at 12 weeks post-treatment. Predictably, sleep efficiency and average total sleep time also increased, which may have played a role in performance improvements. However, 5 mg melatonin supplementation (or 25 mg amitriptyline, to which it was compared) did not contribute to significantly improved sleep or neuropsychiatric parameters when compared to baseline in a double-blind randomized crossover trial with seven adult males who had suffered from post-TBI sleep disorders [15]. Interestingly, patients did self-report increased alertness when on melatonin, and effect sizes revealed improved sleep alertness, duration, quality, and latency (the same effect size patterns were seen with the amitriptyline treatment, in addition to patient self-reports of improved sleep duration and latency). While this study’s small sample size precludes definitive conclusions, it highlights the complexity of identifying the potential roles and physiological mechanisms of melatonin in restoring cognition following TBI.

Moreover, most of the studies highlighted in this review focus on mice and rat models, which were sacrificed within a short period of time after the induction of TBI and subsequently did not produce adequate data to draw conclusions on melatonin’s long-term effects after TBI. In addition, animal models may not be adequate predictors of outcomes in human subjects. Existing melatonin supplementation studies in human TBI patients are sparse, and those that exist are limited by small sample sizes and unclear significance. Therefore, there is a need for longitudinal retrospective studies on human subjects to determine whether melatonin therapy can produce significant differences in functional outcomes after TBI. Longitudinal studies can also be used to better characterize the progress of neuroinflammatory damage and recovery with the use of serial biomarkers and neuroimaging. Biomarkers such as levels of tau, p-tau, amyloid-beta, matrix metalloproteinases (MMPs), glial fibrillary acidic protein (GFAP), nerve fiber layer (NFL), and miRNAs offer some useful indicators of inflammation that can be measured in biofluids, while cerebrovascular dynamics, damage, and impaired metabolic clearance can be monitored over neuroimaging. Useful methods that will be invaluable to the longitudinal characterization of neurodegeneration include MRI to monitor vasoreactivity, blood flow, blood-brain barrier (BBB) permeability, and hypoperfusion; and PET to monitor hypometabolism and impaired metabolite clearance. Animal model studies support that melatonin treatment after TBI significantly improves cognition and behavioral outcomes. However, clinical studies with human subjects are scarce. Considering melatonin therapy’s promising preclinical data, favorable safety profile, and accessibility, further studies are warranted to assess the effects of melatonin as a post-TBI therapy on human subjects. 

## Conclusions

The majority of findings from the studies gathered in this review demonstrate in murine models that melatonin exhibits neuroprotective effects through its antiinflammatory and antioxidant function, making it possibly beneficial in reducing the reactive processes that occur after TBI in the human brain. The proposed mechanisms by which melatonin exerts neuroprotection involve its ability to attenuate proinflammatory NFkB signaling, scavenge free radicals, decrease apoptotic cell death, and reduce the expression of abnormal proteins such as Aβ and p-tau. A reduction in such secondary injury processes may result in decreased risk of developing neurodegenerative diseases such as Alzheimer’s disease following TBI. Beyond the apparent general antiinflammatory and antioxidant actions of melatonin, a review of the evidence identified some preliminary research that has suggested the significance of melatonin receptors specifically in TBI. While there is some evidence to suggest that melatonin is able to reduce post-TBI cognitive decline as measured by subject performance on memory tasks, there is a lack of longitudinal data on whether melatonin decreases the risk of developing dementia after TBI and further human clinical research is warranted.
